# Malaria diagnosis by PCR revealed differential distribution of mono and mixed species infections by *Plasmodium falciparum* and *P*. *vivax* in India

**DOI:** 10.1371/journal.pone.0193046

**Published:** 2018-03-22

**Authors:** Nisha Siwal, Upasana Shyamsunder Singh, Manoswini Dash, Sonalika Kar, Swati Rani, Charu Rawal, Rajkumar Singh, Anupkumar R. Anvikar, Veena Pande, Aparup Das

**Affiliations:** 1 Division of Vector Borne Diseases, ICMR-National Institute for Research in Tribal Health, Garha, Jabalpur, Madhya Pradesh, India; 2 ICMR-National Institute of Malaria Research, Sector 8, Dwarka, New Delhi, India; 3 Department of Biotechnology, Kumaun University, Nainital, Uttarakhand, India; Université Pierre et Marie Curie, FRANCE

## Abstract

Malaria is a vector-borne infectious disease, caused by five different species of the genus Plasmodium, and is endemic to many tropical and sub-tropical countries of the globe. At present, malaria diagnosis at the primary health care level in India is conducted by either microscopy or rapid diagnostic test (RDT). In recent years, molecular diagnosis (by PCR assay), has emerged as the most sensitive method for malaria diagnosis. India is highly endemic to malaria and shoulders the burden of two major malaria parasites, *Plasmodium falciparum* and *P*. *vivax*. Previous studies using PCR diagnostic assay had unraveled several interesting facts on distribution of malaria parasites in India. However, these studies had several limitations from small sample size to limited geographical areas of sampling. In order to mitigate these limitations, we have collected finger-prick blood samples from 2,333 malaria symptomatic individuals in nine states from 11 geographic locations, covering almost the entire malaria endemic regions of India and performed all the three diagnostic tests (microscopy, RDT and PCR assay) and also have conducted comparative assessment on the performance of the three diagnostic tests. Since PCR assay turned out to be highly sensitive (827 malaria positive cases) among the three types of tests, we have utilized data from PCR diagnostic assay for analyses and inferences. The results indicate varied distributional prevalence of *P*. *vivax* and *P*. *falciparum* according to locations in India, and also the mixed species infection due to these two species. The proportion of *P*. *falciparum* to *P*. *vivax* was found to be 49:51, and percentage of mixed species infections due to these two parasites was found to be 13% of total infections. Considering India is set for malaria elimination by 2030, the present malaria epidemiological information is of high importance.

## Introduction

Vector borne infectious diseases constitute major human health problem, especially in the tropical, sub-tropical and mildly temperate countries of the globe [1]. Among other vector borne diseases, malaria being an age-old disease, is associated with human kind since time immemorial and contributes the most to the global mortality and morbidity [2]. Global malaria incidences have increased by five million in 2016 and mortality remains almost similar, as reported by the World Health Organization (WHO) in 2015 [3]. Moreover, India ranks third with respect to total malaria burden in the world and ranks first (51%) when it comes to global *P*. *vivax* incidences [3]. One of the major reasons for endemicity of malaria is complex interactions among the pathogen, vector and host, influenced by local environmental determinants. Malaria therefore is considered to be a strictly local and focal disease [4]. Furthermore, malaria is unique among other vector borne diseases with respect to pathogens; wherein five different species of Plasmodium have been identified to cause malaria in humans. Therefore, to understand malaria epidemiology in a particular endemic location, there is a need to unravel the actual incidences of infection by different species of Plasmodium using a more sensitive diagnostic method (*e*.*g*. PCR assay, see below). This is especially useful in a country where more than one species of Plasmodium is responsible for malaria havoc (*e*.*g*. India).

The various diagnostic tools currently available for identification of Plasmodium species in human blood samples include (i) light and fluorescence microscopy, (ii) immuno-chromatographic lateral flow assays (commonly known as rapid diagnostic tests, RDTs), (iii) serology and (iv) nucleic acid amplification techniques (NATs) that include PCR and isothermal amplification [5]. Other known techniques for identification of Plasmodium species are (v) Loop mediated isothermal AMPlification (LAMP) [6], (vi) flow cytometry [7] *etc*. Out of these tests, the most commonly performed diagnosis of malaria at the primary health care levels is limited to either microscopy or RDT. Whereas the gold-standard microscopic detection of malaria parasites have several limitation in proper diagnosis of malaria [8], in case of RDT, the dye-labelled antibody binds to a parasite antigen, and thereafter the resultant complex is captured on the strip by a band of bound antibody that forms a visible line. Like microscopy, RDT has limitations [9], especially in term of differential sensitivity in different products [10]. However, in comparison to microscopy, RDT is more sensitive in diagnosing malaria parasites in clinical settings [11]. In recent years, the Polymerase Chain Reaction (PCR) technique in amplifying the *Plasmodium* spp. 18S rRNA genes [12] was found to be superior to the traditional microscopy [13] and RDT [14] in detecting malaria parasites. Since molecular biological setup in clinical settings is not always feasible, thereby PCR diagnosis is currently limited to laboratory-based diagnosis [15] and serves as a useful tool for epidemiological understanding of malaria infections.

To this respect, according to the World Health Organisation (WHO), 80% of *P*. *falciparum* incidences and 60% of death due to *P*. *falciparum* infections outside Africa was contributed by India, making the country rank third in the global mortality due to malaria in 2016 [3]. The inimitable climatic condition, topography and malaria vector diversity in India offer a hospitable environment for growth and proliferation of malaria parasites [16]. Malaria is majorly contributed by *P*. *falciparum* and *P*. *vivax*, distributed roughly in equal proportion but not uniform across Indian localities [17]. This situation provides an opportunity for the occurrence of mixed species infection due to these two species [4]. In fact, occurrence of mixed species infections due to these two species is very common and has been reported from different parts of India [17–21]. Since field diagnosis of malaria at the primary health care level in India is majorly performed by microscopy and RDT, and due to low sensitivity/accuracy in diagnosing multiple species with these two methods, cases of mixed species infection are usually missed. Such discrepancy has been recently reported [21], where approximately 17% of mixed infections were initially identified as mono infections due to *P*. *falciparum*. This indicates that misdiagnosis is widely prevalent, which puts a great challenge in proper treatment of malaria in India.

In order to have a larger picture on the distributional prevalence of both mono and mixed infections by the two major malaria parasites (*P*. *falciparum* and *P*. *vivax*) across Indian localities than before, we have sampled 2,333 malaria symptomatic individuals from 11 different locations in nine different states covering almost the entire malaria endemic regions in India. Although we have adapted three different common procedures of malaria diagnosis (microscopy, RDT and PCR assays), we have utilized the most sensitive method (PCR diagnostic assay) to infer the distributional prevalence of both mono and mixed infections due to two most common malaria parasites, *P*. *falciparum* and *P*. *vivax* across different Indian locations.

## Materials and methods

**Fig 1:**
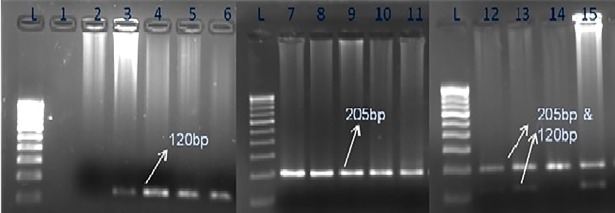
Agarose gel electrophoresis pictures showing bands of 15 representative PCR products. The first lane (lane L) contains ladder (100 bp marker) for comparison of PCR products and for determination of product size. The second lane (Lane 1) contains PCR products from negative control, lane 2 contains product of negative control with human DNA. Lanes 3–6 display 120 bp PCR product signifying *P*. *vivax* mono infection and lanes 7–11 show 205 bp PCR product testifying mono infection of *P*. *falciparum*. Lanes 12–13 and 15 present both the bands of 120 bp (*P*. *vivax*) and 205 bp (*P*. *falciparum*) size in a single sample, indicating mixed species infections due these two species of malaria parasites.

A total of 2,333 finger-pricked blood samples were collected from 11 different collection sites from a period of 2012 to 2015 (Table 1). The sample collection sites were selected based on previous report on the malaria endemicity in that particular Indian state and locations, such as; Delhi [22], Diphu and Guwahati from Assam [23], Shankargarh from Uttar Pradesh [24], Nadiad from Gujarat [17], Betul from Madhya Pradesh [25], Kendujhar [26] and Rourkela [27] from Odisha, Gadchiroli from Maharashtra [28], Chennai from Tamil Nadu [29] and Mangaluru from Karnataka [30]. In particular, due care has been taken to sample from areas reportedly dominated by either *P*. *falciparum* or *P*. *vivax*, and places from where no reports on the incidences of mixed parasitic infections due to these two species of malaria parasites is available previously. Adopting such an approach, we propose to cover all the differential endemic areas in India due to these two species of malaria parasites (e.g., low, middle and high endemic), so that a comprehensive information on the occurrence of different species of malaria parasites in Indian context could be presented. From each malaria-suspected patient, finger-prick blood sample was collected only once (2–3 drops) and was utilized for all the three diagnostic assays. In the field, bivalent RDT kit, Falci-Vax (Zephyr, Biomedical) was used for identification of either single or mixed infections due to *P*. *falciparum* and *P*. *vivax*. Simultaneously, a single blood drop was used for preparation of thick and thin smears on a glass slide for microscopical examinations for the presence of different species of malaria parasites in the laboratory. The Giemsa-stained thick and thin blood smears were visualized under light microscope following the standard protocol by the expert technicians of the National Institute of Malaria Research (NIMR), New Delhi for the identification of various stages of these two species of malaria parasite. Using the rest of the blood drops, 3–4 spots were placed on the Whatman filter paper for subsequent molecular analyses by PCR (see below) at the Evolutionary Genomics and Bioinformatics laboratory of the ICMR- National Institute of Malaria Research (NIMR), New Delhi. All the necessary clearances have been obtained from the human ethics committee of NIMR and written informed consents were taken from all the patients at the time of sample collection. The NIMR human ethics committee has specifically approved this study. Blood spots on Whatman filter paper (3–4 spots) were dried and utilized for molecular diagnostic assay in the laboratory. For this, genomic DNA was extracted from these spots collected on the Whatman filter paper using QIAamp mini DNA kit (Qiagen, Germany) and eluted in 100 μl elution buffer. Nested PCR amplification [13] was performed as follows; in the first step of nested PCR, a pair of Plasmodium genus-specific primers [rPLU5 (5’CCTGTTGTTGCCTTAAACTTC3’) and rPLU6 (5’TTAAAATTGTTGCAGTTAAAACG3’)] were used, which amplified a 1100-bp PCR product from the rRNA small subunit gene (18S rRNA). Following this, the second step uses two species specific primers; *P*. *falciparum* [rFAL1 (5’TTAAACTGGTTTGGGAAAACCAAATATATT3’) and rFAL2 (5’ACACAATGAACTCAATCATGACTACCCGTC3’) and *P*. *vivax* primer pairs [rVIV1 (5’CGCTTCTAGCTTAATCCACATAACTGATAC3’) and rVIV2 (5’ACTTCCAAGCCGAAGCAAAGAAAGTCCTTA3’)] for amplifying specific gene products for these species. Whereas a 120-bp PCR product of Plasmodium 18S rRNA indicates *P*. *vivax* infection, a 205-bp amplified product indicates *P*. *falciparum* infection (Fig 1). The samples harboring mixed infection display bands at both the positions (Fig 1). Detection of *P*. *malarie* and *P*. *ovale* infection by PCR assay following described protocols [20] initially for samples from Betul (Madhya Pradesh), Shankargarh (Uttar Pradesh), Rourkela and Kendujhar (Odisha) (659 samples in total) turned out to be negative for these two parasites, therefore, PCR diagnostic assays for these two parasites were not conducted in the rest of the samples from other seven locations.

**Table 1:** Details of locations (with population coordinates) of 11 malaria sample collection sites from nine different states in India with results on three different diagnostic tests (microscopy, RDT and PCR assay). To be noted that data from different time points of collection from a single collection site have been collated and considered as total number of sample from that particular population.

State	Location of Sample Collection	Abbreviated sample location	Population Coordinates	Time of Sample Collection	Total Sample	Microscopy			RDT			PCR Assay		
						*Pf*	*Pv*	Mixed	*Pf*	*Pv*	Mixed	*Pf*	*Pv*	Mixed
Delhi	Delhi	DEL	28°N	August, 2014	16	0	10	0	0	14	0	0	11	0
			77°E											
Assam	Diphu	DIP	26° N	June-July 2013	79	4	2	0	5	5	0	5	4	0
			93° E											
	Guwahati	GUW	26°N	July, 2013	155	9	15	0	10	20	0	9	16	0
			91° E											
Uttar Pradesh	Shankargarh	SHA	25° N	September, 2015	344	24	83	2	17	96	4	28	91	9
			82° E	October, 2015	570	42	77	2	43	73	2	58	75	6
Gujarat	Nadiad	NAD	23° N	June, 2015	28	3	19	0	4	24	1	2	21	1
			73° E											
Madhya Pradesh	Betul	BET	22° N	September 2012	64	5	30	1	8	23	0	2	26	7
			77° E	July, 2013	355	41	26	6	52	14	9	45	18	12
Odisha	Kendujhar	KEO	22° N	Feburary 2013	100	3	15	0	4	16	0	3	17	1
			86° E											
	Rourkela	ROU	22° N	July- August 2012	140	62	18	7	72	14	9	69	13	12
			85° E											
Maharashtra	Gadchiroli	GAD	19° N	Feburary 2012	118	66	32	17	73	9	22	64	12	40
			72° E	December, 2012	34	29	0	0	28	0	0	27	0	1
Tamil Nadu	Chennai	CHE	13°5’N	June, 2014	41	0	18	0	0	19	0	0	21	0
			80° E											
Karnataka	Mangaluru	MAN	13˚ N	March, 2014	289	36	53	7	39	53	11	38	47	16
			74° E											
**Total**					**2333**	**324**	**398**	**42**	**355**	**380**	**58**	**350**	**372**	**105**
						**764**			**793**			**827**		

For each sample, PCR amplifications were carried out in a final volume of 25 μl, which included 10 pmol of each primer, 0.2 mM of dNTPs, 1 unit of Taq DNA polymerase (Banglore Genei), 1X Taq DNA polymerase buffer and 1μl of DNA. Cycling conditions for the first step included an initial denaturation at 95°C for 5 min followed by 30 cycles of 1 min denaturation at 94°C, 2 min annealing at 60°C and 2 min of extension at 72°C followed by 10 min final extension at 72°C. The cycling conditions for second step included an initial denaturation at 95°C for 5 min followed by 30 cycles of 1 min denaturation at 94°C, 2 min annealing at 55°C and 2 min of extension at 72°C followed by 10 min final extension at 72°C. After PCR amplification, 5 μl of PCR amplified product was electrophoresed on a 2% agarose gel and compared with a 100-bp ladder (Merck) to confirm the amplicon size. To identify false positive results and to check the primer specificity, two negative controls were used, one lacking DNA and the other one containing the genomic DNA of a healthy individual from Delhi. For further confirmation, PCR amplified products (10 each showing single bands for *P*. *falciparum* and *P*. *vivax*; and 10 displaying both the bands) were purified using the ExoSap (Fermentas,), and finally utilized for performing DNA sequencing using Big Dye Terminator Chemistry in a 96-capillary DNA analyzer (ABI 3730XL) under the facility of ICMR-NIMR, New Delhi.

## Results

**Fig 2:**
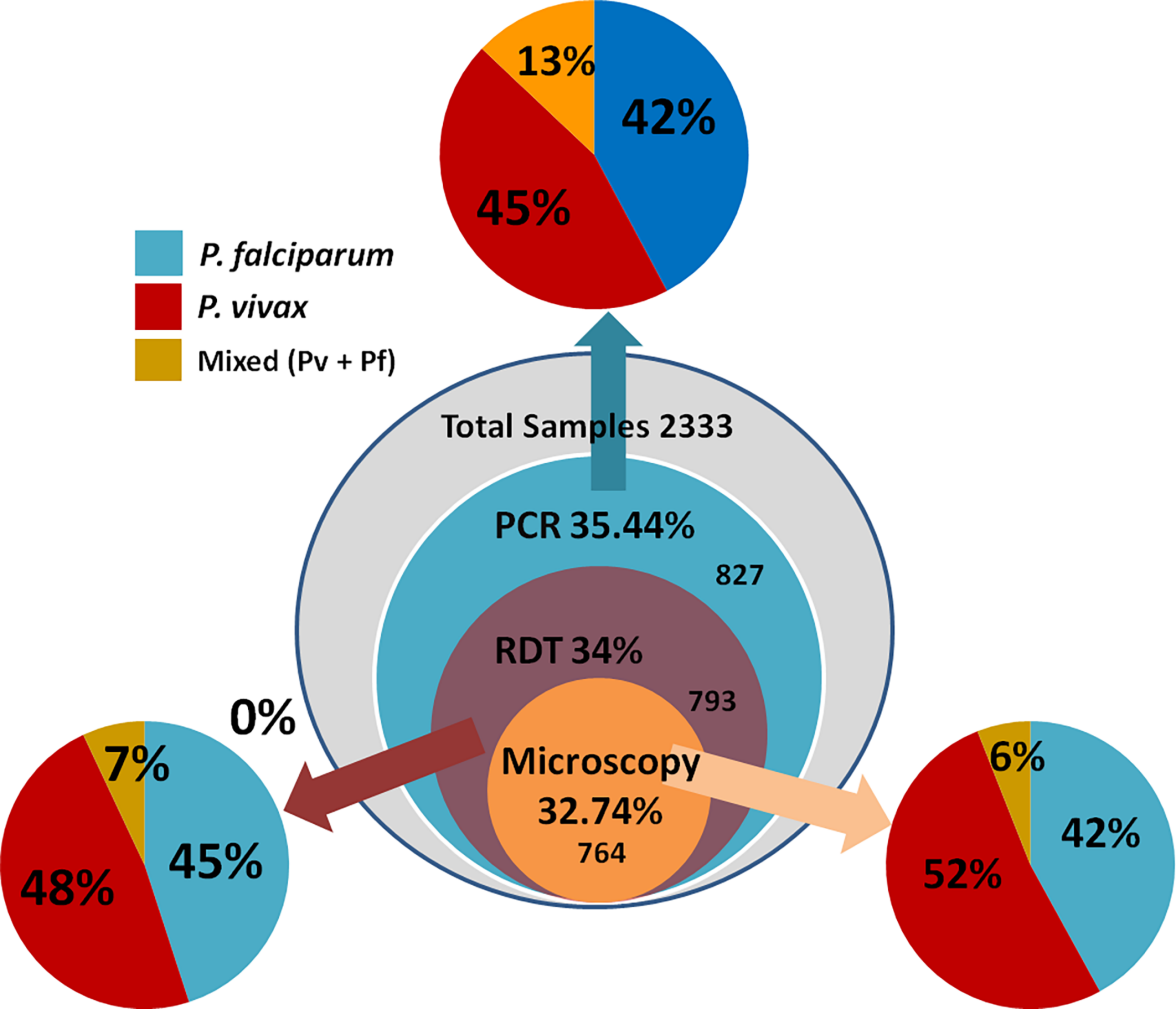
Visual representation on the comparative assessment (in number and percentage) of efficacy of three different malaria diagnostic methods (microscopy, RDT and PCR assay). To be noted that out of the total 2333 collected malaria-symptomatic individuals (outer-most circle of the middle circles, in grey), PCR assay could identify 827 (35.44%) as positive for malaria parasite infection out of which 42% was *P*. *vivax*, 45% *P*. *falciparum* and 12.69% mixed infection due to these two species. In comparison, the RDT (third circle from out) and microscopy (4th circle from out) could identify less number of infections.

In the present study, 2333 blood samples from malaria symptomatic individuals were collected at different time period (February 2012 to October 2015) adopting both active and passive surveillance methods. Malaria symptomatic individuals from as many as 11 different geographical locations (encompassing nine states) spanned vertically from Delhi in North and Tamil Nadu in the South and horizontally from Gujarat in the west and Asom in the east were collected, thus covering almost all the malaria endemic regions in India (Table 1) in the present study. Malaria diagnosis of all these samples was performed by three different methods (Microscopy, bivalent RDT kits and PCR,) to characterize the samples based on the type of infection caused by either single (mono) infection of either *P*. *falciparum* or *P*. *vivax*, or mixed species infections by these two species. Out of the 2,333 samples, microscopic examination resulted in 764 malaria positive cases (32.74%), of which 324 (42%) as *P*. *falciparum* mono infections, 398 (52%) as *P*. *vivax* mono infection and 42 (6%) as mixed infection (Fig 2). At the same time, bivalent RDT kit reported 733 positive malaria cases (31.41%); 355 (45%) *P*. *falciparum* mono infection, 380 (48%) *P*. *vivax* mono infection and 58 (7%) as mixed infection (Fig 2). However, PCR assay could detect 827 positive cases for malaria parasite infection (35.44%); of which 350 (42%) as *P*. *falciparum* mono-infection, 372 (45%) as *P*. *vivax* mono infection and 105 (13%) as mixed species infection (Fig 2). As a confirmation measure of the PCR diagnostic assay in term of mixed infections, the gel bands were cut for the two species and sequenced and successfully aligned with the respective reference sequences of each species (*P*. *falciparum* and *P*. *vivax*). The newly generated DNA sequences have been submitted to the GenBank with accession number MG708202 –MG708221. The detail number of mono infections by the two parasites and also mixed infections by both these parasites in a single sample and the comparative assessment among all the 11 population samples have been provided in Table 1. If all the three diagnostic methods were to be graded based on sensitivity, PCR diagnostic method was found to be the most efficient (35.44%), followed by RDT (34.0%) and microscopy (32.74%) (Fig 2). Therefore, we utilized the data on PCR diagnostic assay for further analyses to comprehend distributional prevalence of different types of infections (mono and mixed species) in different geographical locations due to *P*. *falciparum* and *P*. *vivax* in India (see below). Considering both the mono and mixed infection detected with PCR diagnostic assay, 455 individuals were found to be infected with *P*. *falciparum* and 477 with *P*. *vivax*. Therefore, the proportion of *P*. *falciparum* to *P*. *vivax* infection from this study amounts to 49:51.

**Fig 3:**
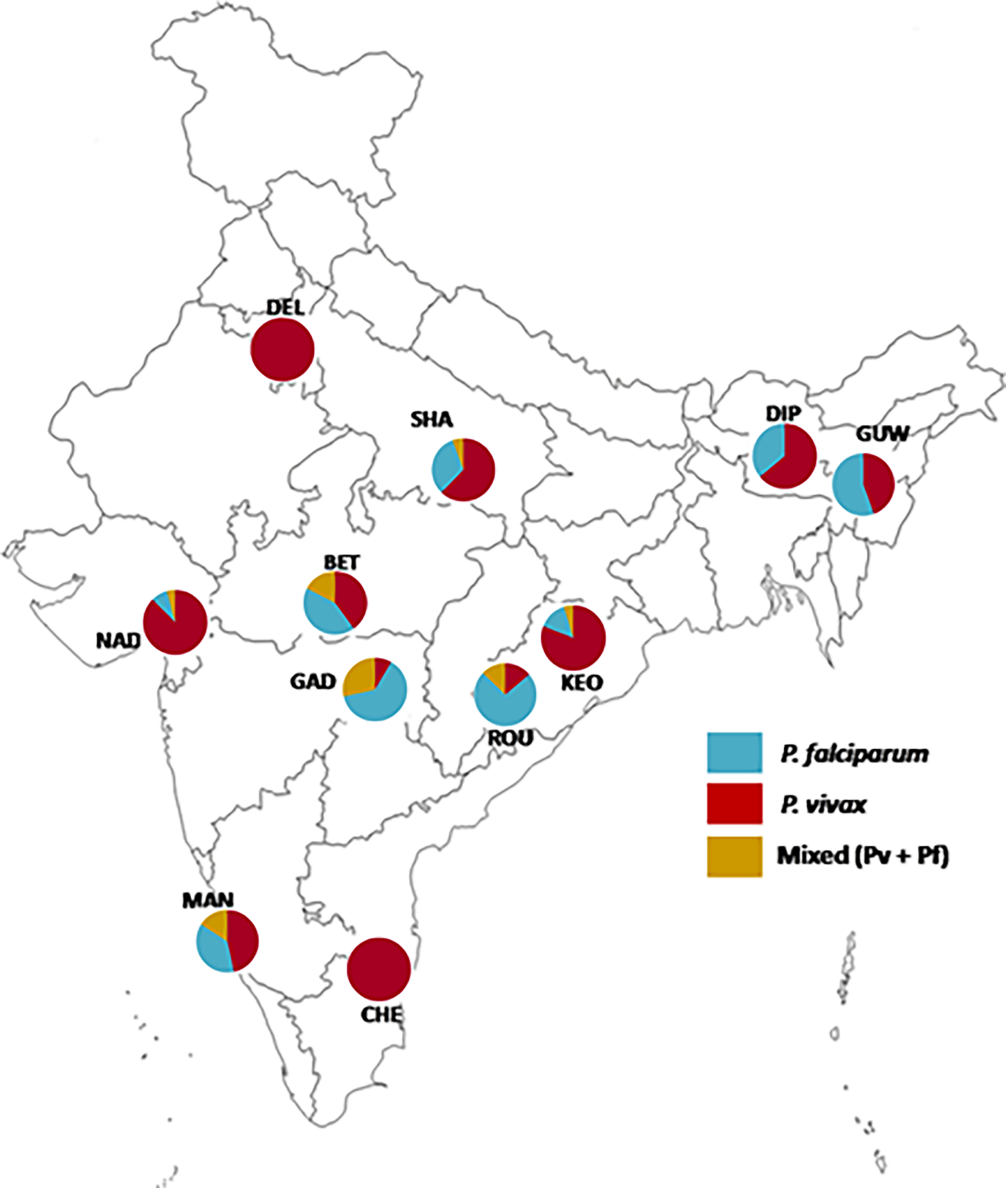
Map of India showing malaria sample collection site. Each site is represented by a pie-chart three different kinds of infection (two types of mono infections and a mixed species infection due to *P*. *falciparum* and *P*. *vivax*). To be noted here that locations in all the four directions (peripheral populations) (north, east, west and south) are majorly dominated by *P*. *vivax*, but in northeast, south-west and middle Indian locations *P*. *falciparum* was found to be in higher abundance than *P*. *vivax*. Mixed parasitic infections majorly are restricted to middle of India.

Since the present study is based on a large sample size covering almost all the malaria endemic locations in India, geographic structure on the distribution of different types of infections due to *P*. *falciparum* and *P*. *vivax* (mono or mixed) could be apprehended. Both the northern and southern Indian samples (DEL and CHE) were found to be under the sole dominance of *P*. *vivax*—not a single case of either *P*. *falciparum* mono or mixed infection could be detected (Table 1; Fig 3). Very similarly, majorities of infections in western (NAD) and eastern population samples (KEN) were found to be subjugated by *P*. *vivax*. Similar dominancy of *P*. *vivax* was also seen in malaria samples from MAN (south west), GUW (northeast) and SHA (north). However, like *P*. *vivax*, absolute mono infection of *P*. *falciparum* could not be detected from any population sample, but in three locations (BET, ROU and GAD), dominancy of *P*. *falciparum* mono infection over the two other types of infections (*P*. *vivax* mono and mixed infections) could be noticed, confirming wide prevalence of *P*. *falciparum* mono infections in the central regions of India (Fig 3). Incidentally, in all samples from two locations (DIP and GUW) from Asom (north-eastern India), mono infections of either *P*. *falciparum* or *P*. *vivax* could only be observed. The results thus indicate regional biasness on the prevalence of mono infections due to *P*. *falciparum* and *P*. *vivax* in India (Fig 3).

Interestingly, wide prevalence of mixed infections was found in Indian locations, and with considerably high proportions (13%). With close observation on the distributional prevalence of mixed species infections, it was noted that mixed species infections are common in the central Indian locations (where *P*. *falciparum* is dominant over *P*. *vivax*). For example, the highest proportion of mixed infection due to *P*. *vivax* and *P*. *falciparum* was found in a central Indian population, sampled from GAD (29%), followed by BET (17%). Incidences of mixed species infections were also found to be high in a south-west coastal city (MAN, 16%). Surprisingly, not a single case of mixed infection could be detected in the north eastern state of Asom, even though mono infections due to both *P*. *falciparum* and *P*. *vivax* are widely prevalent (Fig 3). The results therefore indicate that like the distribution of mono infections due to *P*. *falciparum* and *P*. *vivax* (see above), regional biasness well exists on the prevalence of mixed malaria parasite infections due to these two species.

## Discussion

The present study is by far the largest epidemiological investigation covering almost all the entire malaria endemic areas of India (nine states, 11 locations, covering north to south, east to west) in unravelling differential prevalence of mono infections by *P*. *falciparum* and *P*. *vivax* and also mixed infections by these two species of malaria parasite in a single study. Other previous studies have either covered some restricted areas of India [20] or have considered low number of samples [18]. For diagnosis of malaria parasites, we have followed traditional (microscopy), antigen-based (RDT) as well as molecular (PCR assay followed by DNA sequencing) methods. Among all the methods, PCR assay turns out to be the most sensitive method to detect either mono or mixed parasite infections; as both microscopy and RDT has failed to diagnose the presence of malaria parasites that otherwise PCR could. This is evidenced by the fact that microscopy failed to detect 63 positive cases and RDT failed to detect 34 positive cases as compared to PCR assay.

There might be several factors by which microscopy might have failed to detect some of the infections. For example, in the present study, microscopy has misdiagnosed several mono *P*. *falciparum* and mixed cases as mono *P*. *vivax* case. This can be ascribed to certain facts like (i) time of collection of blood sample, (ii) availability of all the life-cycle stages of *P*. *vivax* in peripheral blood samples in comparison to only ring and gametocyte stages of *P*. *falciparum* [18] (iii) similar morphology of ring stages of both *P*. *falciparum* and *P*. *vivax* [31] and (iv) abundance of *P*. *vivax* gametocytes in comparison to *P*. *falciparum* [32]. Furthermore, it is known that (v) *P*. *vivax* can increase deformability of infected RBCs more effectively than *P*. *falciparum* [33], making it more prominent to detect under microscope. Very similarly, RDT has misdiagnosed many mixed infection cases, as *P*. *vivax* mono infections and has also failed to detect few mono *P*. *falciparum* cases. Since the RDT kit (Falci-vax by Zephyr) that we used here is based on histidine rich protein (hrp2) for diagnosis of *P*. *falciparum* and Lactate dehydrogenase (ldh) protein for diagnosis of *P*. *vivax*, and deletion and mutations are commonly reported in the *Pfhrp-2* gene [34], misdiagnosis is inevitable. Taking all these aspects into consideration, PCR assay is considered to be the most ideal method for identifying mixed infections [35] as well as mono infection by *P*. *falciparum* and *P*. *vivax* [18, 20] that otherwise would be overlooked using classical diagnostic methods.

Differential distributional prevalence of mono infections of *P*. *falciparum* and *P*. *vivax* in India is quite interesting, as it provides a unique epidemiological understanding on malaria in India. Out of the 827 malaria positive samples, 455 were found to be infected with *P*. *falciparum* and 477 with infection by *P*. *vivax* with a proportion of 49:51. Traditionally, with regard to malaria burden caused by the two-different species of malaria parasites (*P*. *falciparum* and *P*. *vivax*), India is known to be majorly burdened with *P*. *vivax* malaria (3), but over time there has been a dramatic shift from *P*. *vivax* to *P*. *falciparum* malaria. The ratio of *P*. *falciparum vs*. *P*. *vivax* malaria was 0.41 in 1985 which had drastically shifted to 1.01 (in the ratio of 50:50) in 2007 [36]. The results obtained here therefore, are in corroboration of the previous reports on the shifting paradigm of overall dominance of *P*. *falciparum* over *P*. *vivax* in India [4, 16]. However, there is regional biasness on the overall dominance of these two species in India. As reported before, *P*. *vivax* continues to be largely prevalent in specific Indian regions (*e*.*g*. northern and southern parts of India) where it is highly dominant [23, 37]. In addition, to our surprise, not a single case of either mono or mixed infection involving *P*. *falciparum* could be detected in either the northern or the southern Indian locations. Whether this observation will hold true with a much larger sample size is not known, as the present study is restricted to only Delhi (northern India) and Chennai (southern India) and that too with a limited sample size with non-uniformity of time of sample collection in all the locations. Furthermore, *P*. *vivax* is also appreciably prevalent in the north-eastern and western Indian locations (Fig 3), However, *P*. *falciparum* was found to be dominant over *P*. *vivax* in the eastern, northeastern and central parts of India. This information is not new, as the eastern and north-eastern belts are known to be under the clouds of *P*. *falciparum* [38, 39]. To add to this, according to WHO [3] and as reported in several epidemiological studies [4, 16], the highest malaria endemic state of India is Odisha. This state being the epicenter of malaria infection in India, also possess high diversities in species of malaria parasites [40, 41], high diversity of different antigenic [42] and drug-resistant [43] genes, with highest malaria incidence and deaths in comparison to other states of India [44, 45]. Similar was the case here; samples from two locations from Odisha (ROU and KEN) possess the highest diversity of both mono and mixed parasite infections, corroborating the earlier observation. The high incidences of *P*. *falciparum* malaria contributed by the eastern and middle part of India therefore overshadow the *P*. *vivax* malaria found in high prevalence in the northern and southern India to bring the ratio of *P*. *falciparum* to *P*. *vivax* malaria to 49:51 at Indian level.

Out of the 827 malaria positive samples diagnosed by PCR, about 13% were found as mixed infection due to *P*. *falciparum* and *P*. *vivax*. Mixed species infections due to *P*. *falciparum* and *P*. *vivax* are majorly prevalent in the middle and southwest coast of India, with mild prevalence in other locations (Fig 3). Interestingly, no case of mixed infection could be found in the north-eastern regions of India. It is considered that the number of mixed infection cases is usually under-reported in clinical and epidemiological studies by less than 2% of the actual prevalence [31]. Also, cases of mixed malaria infection have regularly been reported in many tropical malaria endemic countries like Thailand, Cambodia, Papua New Guinea, India and many more [21, 46–48]. In a global comparison on the distributional prevalence of mixed malaria species infection, Southeast Asia alone was found to contribute to about one-third cases of mixed malaria infections found in the global scale, whereas, Africa contributed comparatively lesser number [49]. Since malaria in Europe is majorly known as imported malaria, number of mixed parasite infections was reported to be very similar to the incidence found in West Africa [50]. However, in Australian continent, incidences of mixed malaria infections are regularly reported from Papua New Guinea [51]. Such wide-spread global prevalence of mixed malaria parasite infection might cause serious implications on malaria public health, as treatment and control of one parasite have an effect on the clinical epidemiology of the sympatric species [52]. For example, cases of recurrence of *P*. *vivax* malaria after *P*. *falciparum* treatment have been reported in Southeast Asian countries [53], [54]. Moreover, since mixed parasite infections are often associated with severe malaria [55, 56] and India reports cases of severe malaria due to both *P*. *falciparum* and *P*. *vivax* [4], the importance of mixed malaria infections in public health should be taken seriously and appropriate treatment/control measures are to be kept in place in India.

The present study involving large number of malaria samples covering nine different Indian states could unravel epidemiological information on the distributional prevalence of mono as well as mixed parasite infections in India. The results highlight wide distribution of mono infections of *P*. *falciparum* as well as *P*. *vivax* along with a high proportion (13%) of mixed infection due to these two species. It was interesting to note that *P*. *vivax* is majorly distributed at the peripheral populations in each direction (north, south, west and to some extent, east), whereas, *P*. *falciparum* is restricted to north-east, east, central and southwestern regions of India. Incidentally, major incidences of mixed infections take place in the central regions of India (Fig 3). However, our study is limited to only nine states of India, and no information on the distributional prevalence of either mono or mixed parasite infections following PCR assay in other states reporting malaria (*e*.*g*. Punjab, Haryana, Kerala *etc*.) is available yet. Since mixed infection cases put huge malaria burden, many more studies (with large sample size) are to be carried out in other parts of India that are known to be endemic to both *P*. *falciparum* and *P*. *vivax*. Furthermore, *P*. *malariae* is emerging as a potential threat and *P*. *ovale* cases are emerging in recent years across the malaria endemic regions of the globe [57, 58]. In India also, these two species of malaria parasites are reportedly in the process of expanding their range [59].

Historically, India has experienced the burden of malaria since time immemorial. Although malaria incidences were very high before coordinated interventions by the government, the launch of the National Malaria Control Program (NMCP) resulted in a drastic drop of malaria cases to less than 50,000 with no reported malaria mortality in 1961. However, malaria staged a dramatic comeback, with 6.45 million estimated malaria cases in 1976 [60]. Nevertheless, renewed governmental efforts did control malaria to a greater extent that was evidenced by a downward trend of reported cases, which is continuing till date. India has recently launched National Framework for Malaria Elimination (NFME) to achieve malaria elimination by 2030 [61]. To this extent, a recent meta-analyses of reported incidences of different infections by all the five malaria parasites in India by PCR diagnostic assay has unraveled several interesting facts that might hinder the targeted malaria elimination program in India [60]. Moreover, considering the complexity of malaria that India possess [17] together with several distinctive features of Indian malaria parasites [4], malaria control/elimination program could only be successful if data on the variable incidences of infections by different malaria parasites (as presented here) are generated and considered in the malaria elimination program. Such understanding will also aid in developing novel early warning system for malaria outbreak prediction [62, 63] and eventually to the malaria elimination program.
